# Supplementary material for: Mechanisms governing the pioneering and redistribution capabilities of the non-classical pioneer PU.1
Source: Nat Commun. 2020 Jan 21;11:402. doi: 10.1038/s41467-019-13960-2 (PMC6972792; doi:10.1038/s41467-019-13960-2)
Supplement: Supplementary file 7 — Source data [file 41467_2019_13960_MOESM7_ESM.zip › Source_Data/Figure6/Figure6B_MotifScanOutput/FLI1_mutPU.1/homerResults/motif4.similar.html]

motif4

## Information for motif4

G
A
T
C
C
A
T
G
C
A
T
G
T
C
G
A
A
C
G
T
A
C
T
G
G
C
T
A
A
T
C
G
C
G
A
T
T
A
G
C
C
G
T
A
A
G
T
C
  
Reverse Opposite:  

T
C
A
G
A
C
G
T
A
T
C
G
G
C
T
A
T
A
G
C
C
G
A
T
T
G
A
C
G
T
C
A
A
G
C
T
G
T
A
C
G
T
A
C
C
T
A
G
  

|  |  |
| --- | --- |
| p-value: | 1e-218 |
| log p-value: | -5.036e+02 |
| Information Content per bp: | 1.617 |
| Number of Target Sequences with motif | 1042.0 |
| Percentage of Target Sequences with motif | 10.16% |
| Number of Background Sequences with motif | 929.8 |
| Percentage of Background Sequences with motif | 2.44% |
| Average Position of motif in Targets | 98.6 +/- 52.2bp |
| Average Position of motif in Background | 101.3 +/- 58.0bp |
| Strand Bias (log2 ratio + to - strand density) | -0.0 |
| Multiplicity (# of sites on avg that occur together) | 1.07 |
| Motif File: | file (matrix) reverse opposite |

### Similar de novo motifs found

|  |  |  |  |  |  |  |  |
| --- | --- | --- | --- | --- | --- | --- | --- |
| Rank | Match Score | Redundant Motif | P-value | log P-value | % of Targets | % of Background | Motif file |
| 1 | 0.980 | A T G C A T C G C A T G T G C A A C G T C T A G G C T A A T C G C A G T A T G C G T C A | 1e-215 | -495.974917 | 10.73% | 2.77% | motif file (matrix) |
| 2 | 0.980 | T A C G T C G A A C G T A C T G C G T A A T C G A C G T A G T C C G T A A G C T | 1e-213 | -491.449777 | 9.79% | 2.32% | motif file (matrix) |
| 3 | 0.960 | A G C T A C T G G C T A T A C G A C G T T G A C C G T A A G C T | 1e-210 | -484.469853 | 14.66% | 5.06% | motif file (matrix) |
| 4 | 0.977 | C A T G T A C G A C G T A C T G C G T A A T C G A C G T A G T C C G T A A T G C G A T C A G T C A G T C A G T C | 1e-209 | -482.496316 | 9.34% | 2.14% | motif file (matrix) |
| 5 | 0.980 | A G C T C A T G G C T A T A C G C A G T G T A C T C G A A G C T G A T C | 1e-205 | -474.289458 | 11.30% | 3.19% | motif file (matrix) |
| 6 | 0.967 | C A G T T C A G A C G T A C T G C G T A A T C G A C G T A G T C C G T A A T G C G T C A G A C T T A C G | 1e-199 | -460.261677 | 9.41% | 2.28% | motif file (matrix) |
| 7 | 0.938 | A C G T A C T G C G T A A C T G A C G T A G T C C G T A | 1e-194 | -448.255031 | 10.30% | 2.80% | motif file (matrix) |
| 8 | 0.874 | C G A T C A T G G T C A T C G A A C G T A G T C C G T A | 1e-166 | -383.182444 | 18.09% | 8.20% | motif file (matrix) |
| 9 | 0.758 | G C A T A C G T T G C A T C A G A G C T G T A C C T G A T G A C G C T A T A G C | 1e-62 | -143.446191 | 2.77% | 0.62% | motif file (matrix) |
| 10 | 0.761 | T G C A A C G T A C G T C G T A A C T G A C G T A G T C C G T A A G T C | 1e-23 | -55.104170 | 1.43% | 0.43% | motif file (matrix) |
